# Parental opinions regarding consent for observational research of no or minimal risk in the pediatric intensive care unit

**DOI:** 10.1186/s40560-019-0411-3

**Published:** 2019-12-16

**Authors:** Jessica Hodson, Christiana Garros, Jodie Jensen, Jonathan P. Duff, Gonzalo Garcia Guerra, Ari R. Joffe

**Affiliations:** 1 Division of Pediatric Critical Care Medicine, Department of Pediatrics, Stollery Children’s Hospital and University of Alberta, Edmonton, Alberta Canada; 2 4-546 Edmonton Clinic Health Academy, 11405 87 Avenue, Edmonton, AB T6G 1C9 Canada; 3 John Dossetor Health Ethics Center, University of Alberta, Edmonton, Alberta Canada

**Keywords:** Consent, Observational research, Pediatric intensive care unit, Survey

## Abstract

**Background:**

The aim of the study was to determine opinions and knowledge regarding the process of obtaining informed consent to participate in observational research in pediatric intensive care.

**Methods:**

Survey 1 asked decision makers what model(s) of consent was acceptable for each type of observational research both before and after background information. Survey 2 asked decision makers about the experience of being asked for consent to observational research, and knowledge regarding the consent process both before and after background information.

**Results:**

Cooperation rate was 100/117 (85%). The proportion in favor of any of the offered alternatives to signed informed consent for observational research, after receiving all the background information, was 74-80%, lowest for observational prospective research with a minimal risk intervention 37/50 (74%; 95% CI 60–84%). The proportion who agreed they felt overwhelmed by being approached for consent to observational research was 26 (52%; 95% CI 39-65%). Most respondents (from 60 to 74%) felt they understood the concepts regarding observational research; however, after reading background information, most (from 60 to 74%) felt their understanding had improved “a great deal”.

**Conclusion:**

Understanding of risk, practical difficulties, consent bias, and Research Ethics Board safeguards was poor. Future study is needed to confirm our finding that most agreed with alternative methods of consent for observational research.

## Introduction

Informed consent to participate in research is morally required to protect patient autonomy to freely determine the cost and benefit balance of their own participation in a research study. As a general standard, for research involving risk to the patient or using confidential information about the patient, this informed consent is given prior to participation in the research study. In children who have never been competent to exercise their autonomy, the legal guardian (usually the parents) has the autonomy to make surrogate decisions for the patient based on that patient’s best interests. In addition to respect for autonomy, there are other considerations regarding the requirement for informed consent that affect the benefit/nonmaleficence balance for the patient from participation in research. Specifically, in observational research that has no or only minimal risk for participants, respecting autonomy by asking for signed informed consent can have adverse effects for the patient, decision maker, and future patients that may not be in the best interests of the patient [1].

A review of the literature regarding observational intensive care unit research of no or minimal risk found several themes regarding adverse effects (i.e., costs) from the requirement for informed consent. Detailed information is given in Additional file 1: Table S1; briefly, themes included: 1) added stress on the decision maker; 2) lack of decision maker knowledge about the research process and safeguards; 3) the importance of consent bias; and 4) the resulting lack of research. Better knowledge of these factors may change the decision maker’s opinion of the cost/benefit balance of requiring informed consent [1–14]. The literature review also found alternative consent models (i.e., alternatives to signed informed consent) for performing observational research in intensive care, which can minimize these problems. Detailed information is given in Additional file 1: Table S2; briefly, models included: a) waived consent; b) opt-out; c) physician consent; and d) broad authorization [7, 12, 13, 15–17]. The acceptability of these alternative models has been variable, and sometimes dependent on factors such as whether research would be impracticable (e.g., no decision maker available), or provision of information about the research process [7, 13, 16].

To our knowledge, the opinions of decision makers for children in the pediatric intensive care unit (PICU) regarding these problems and options are unknown. This study aims to determine a) decision-maker opinions and knowledge regarding the process of obtaining informed consent to participate in observational research of no or minimal risk; and b) decision-maker opinions regarding alternative models for consent to participation in observational research of no or minimal risk, after being given sequential information on the problems with requiring signed informed consent. We hypothesized that decision makers a) find the consent process stressful and lack knowledge of the research process (safeguards, consent bias, and staff burden); and b) when informed of issues involved with the requirement for signed informed consent, to observational research of no or minimal risk, would change their opinions in favor of alternative models for consent.

## Methods

### Ethics approval

This study was approved by the Health Research Ethics Board of the University of Alberta (Pro00057806). Voluntary completion of the survey was considered as consent to participation.

### Questionnaire administration

Parents or legal guardians were approached in the PICU of the Stollery Children’s Hospital to ask for their voluntary participation in the survey studies at a time when their child was clinically stable (i.e., not at a time when family was considered by the research team to be under peak stress). Potential participants were asked if they are “a parent or guardian of a child admitted to the PICU”, and if so, if they would agree to “voluntarily participate in a survey asking about their opinions regarding consent to research study participation”. They were offered a small incentive to participate of a “$5 gift card”. The cover letter stated that “we very much value your opinion on this important issue”, that the survey is anonymous and voluntary, and that return of a survey is considered consent to participate. The time allowed to complete the survey was unlimited, and most were returned hours or even days after agreement to participate. The survey responses were entered into an electronic, secure, survey distribution and collection system (REDCap, Research Electronic Data Capture). There were two surveys, each covering one of the objectives/hypotheses above.

### Questionnaire development

We followed published recommendations for survey methodology [18]. To generate the items for the questionnaires, we searched Medline from 1980 to 2012 for articles about consent to intensive care research. This was followed by collaborative creation of the background section and questions for the surveys by the authors. Content and construct validation was done using a table of specifications (rating the importance of topics included in the surveys, and the adequacy of addressing them) filled out by experts including two intensivist researchers in the Canadian Critical Care Trials Group who have done research on consent in the intensive care unit, and two research coordinators with at least 2 years of clinical research experience in the pediatric intensive care unit. Content validation was done by pilot testing of the surveys: by the experts above, by non-medical parents in the PICU (*n*=2), and by PICU nurses (*n*=2). Each pilot test was followed by a semi-structured interview by one of the authors to ensure clarity, realism, validity, and ease of completion. A published clinical sensibility tool was used for the expert and pilot testing [18]. After minor modifications, the surveys were approved by all the authors.

### Questionnaire content

The surveys both ask about observational research, with the first aiming to determine acceptance of alternatives to signed informed consent after receiving background information, and the second aiming to determine stress associated with being approached for consent and understanding of the background information. We report both surveys here given their related aims.

#### Survey 1 (see Additional file 2)

The background section described the baseline scenario of having a child admitted to and cared for in the PICU, the types of observational research we are referring to (retrospective chart review; prospective chart review; prospective chart review with an intervention of no, or of minimal risk), and the five main types of consent models considered (signed informed consent; opt-out; physician consent; broad authorization; and waived consent) (Table 2). The survey asked demographic questions (age categories; sex; education categories; work in health-care field categories; and having been approached for consent to a research study for their child) and asked what model(s) of consent was acceptable for each type of observational research. Then we sequentially presented three potentially modifying sets of background information (REB process and safeguards; stress on the decision maker; difficulty in obtaining consent as a barrier to research; and consent bias) and asked the same questions about what model(s) of consent was acceptable for each type of observational research. Finally we asked whether the information given in the survey will influence decisions in the future, and asked for free text to the questions: if yes, “in what way”; and if no, “why not?”

#### Survey 2 (see Additional file 3)

The background section described the baseline scenario of having a child admitted to and cared for in the PICU, with a description of being approached for observational research of minimal risk (requiring a cuff blood pressure measurement). The survey asked the same demographic questions; then asked about the experience of being approached for consent to this type of observational study (i.e., stress, emotional burden, anxiety, worry, etc.). Then we asked about knowledge regarding the consent process (i.e., awareness/understanding of independent ethics committees; of no or minimal risk of harm; of safeguards being in place; of consent bias; of difficulty in performing research; etc.). Finally, we asked whether understanding of the relevant consent process improved after a sequence of three sets of background information (defining observational study with minimal risk; consent bias and difficulty obtaining consent; and REB review and safeguards) (Table 2). Response choices were on Likert scales of: “strongly disagree, disagree, neither agree nor disagree, agree, strongly agree”; “did not understand, understand a little, understand most of it, understand very well”; and understanding improved on a scale of 1–5 as “not at all, somewhat, a great deal”, depending on the type of question. Finally, we asked the same question as in survey 1 about whether the information would influence decisions in the future, with the free text questions.

### Statistics

Using REDCap allowed anonymous survey responses to be downloaded into an SPSS database for analysis. The proportions of respondents with different answers are expressed as percentages. As this was exploratory research, and we did not know what to expect for participant responses, we could not estimate a sample size for a reasonable 95% CI around our primary outcomes. Thus, we planned for 50 respondents to each of the two surveys. The primary outcome for Survey 1 was: the proportion (and 95% adjusted Wald confidence interval, 95% CI) in favor of any of the listed alternatives to signed informed consent to observational research of no or minimal risk after receiving all of the background information. Secondary outcomes were the change in the proportions at each stage of information provision, compared using Chi-square statistic, with P≤ 0.05 after Bonferroni correction for multiple comparisons considered significant. The primary outcome for Survey 2 was: the proportion (and 95% CI) who feel overwhelmed on being approached for consent to observational research of no or minimal risk. Secondary outcomes include: the proportion with each response to each question about the level of stress, knowledge of the informed consent process, and possible adverse effects of requiring consent; and understanding each aspect of the consent process after receiving background information. We provide 95% CI for the primary outcomes to report the precision of the estimated response proportion. Post hoc, we examined whether demographic variables were associated with the primary outcomes using Fisher exact tests. The demographic variables were dichotomized into: age <34 years vs. ≥35 years; sex male vs. female; child age <2 years vs. ≥2 years; no degree/diploma vs. any degree/diploma; and previous approach for research no vs. yes.

## Results

### Response rate

Surveys were distributed from November 2015 to April 2016 by research staff when available, and then from July to August 2016 by the summer student. Of 239 eligible patients, 111 were missed (parents not available during screening time, usually due to short admission times of <3 days), 11 were not approached due to social reasons (child was expected to die within 24 h, suspected child abuse, or foster care without a legal guardian present), and 117 were approached for participation. Of these 117, 11 (9%) refused participation, and 6 (5%) did not return the survey, for 100 responses (cooperation rate 100/117, 85%).

### Description of cohorts

The demographics of the 50 respondents to each survey are given in Table 1. Most respondents were age 25–44 years, female, with young (<7yo) children, with at least high school education, and not working in the field of medicine/health. Only 40% (survey 1) and 48% (survey 2) had never been approached for having their child participate in a research study. No guardian did both surveys (i.e., only one of the surveys was done by any individual).

**Table 1:** Demographics of survey respondents

Demographics	Survey 1	Survey 2
Age		
18–24 y	4 (8%)	1 (2%)
25–34 y	24 (48%)	29 (58%)
35–44 y	18 (36%)	13 (26%)
45 y +	4 (8%)	5 (10%)
Sex: female	37 (76%)	34 (69%)
Age of your child		
<2 y	23 (46%)	22 (44%)
2–6 y	11 (22%)	14 (28%)
7–11 y	9 (18%)	7 (14%)
12 y +	6 (12%)	6 (12%)
Level of education		
Not high school	6 (12%)	5 (10%)
High school	9 (18%)	6 (12%)
At least 1 y post-secondary	7 (14%)	11 (22%)
Post-secondary degree/diploma	28 (56%)	27 (54%)
Work in field of medicine and health		
No	41 (82%)	43 (86%)
Yes	9 (18%)	6 (12%)
Physician	0	1 (2%)
Nurse	0	0
Other	9 (18%)	5 (10%)
I have been approached to have my child participate in a research study during this or any other previous hospitalization		
Yes, and I did give consent	24 (48%)	20 (40%)
Yes, and I did not give consent	6 (12%)	5 (10%)
No	20 (40%)	24 (48%)

Note: numbers may not add up to *n*=50 respondents for each survey. This is because a few respondents left the demographic variable response blank. The percentages provided in the table are always of the *n*=50 respondents

#### Survey 1

The results are shown in Table 2. The primary outcome, the proportion of respondents in favor of any of the offered alternatives to signed informed consent to observational research of no or minimal risk after receiving all of the background information, was: for observational retrospective 40 (80%; 95% CI 67–89%); observational prospective 39 (78%; 95% CI 65–87%); observational prospective with no risk intervention 41 (82%; 69–90%); and observational prospective with minimal risk intervention 37 (74%; 60–84%). For each type of observational study, the proportion of respondents finding each method of consent acceptable did not statistically change from before and after each of the series of three sets of background information (Chi-square all *p*>0.15). The proportion of respondents, across all types of observational research and after all background information, was always highest for signed-informed consent (although not by a majority), and lowest for waived consent (although still acceptable for a significant minority). Each type of alternative method of consent was acceptable for about one-third of respondents across all types of observational research, with the exception of waived consent. When asked “do you think the information given in this survey will influence your decision to have your child participate in an observational research study if asked in the future?”, responses were yes for 22 (44%) and no for 26 (52%).

**Table 2:** Survey 1: types of consent acceptable for observational research both before and after reading background information

Type of observational research	Signed informed consent	Opt-out consent	Physician’s consent	Waived consent	Broad authorization consent	Some alternative method	No method
Observational retrospective (e.g., chart review)							
No background	26 (52%)	19 (38%)	17 (34%)	15 (30%)	22 (44%)	41 (82%)	1 (2%)
Background 1	30 (60%)	23 (46%)	12 (24%)	11 (22%)	21 (42%)	36 (72%)	2 (4%)
Background 2	24 (48%)	20 (40%)	13 (26%)	14 (28%)	18 (36%)	45 (90%)	0 (0%)
Background 3	24 (48%)	17 (34%)	16 (32%)	13 (26%)	18 (36%)	40 (80%)	0 (0%)
*P* value	p=0.59	p=0.67	0.65	0.90	0.79	0.15	0.29
Observational prospective (e.g., chart review)							
No background	26 (52%)	18 (36%)	16 (32%)	14 (28%)	21 (42%)	41 (82%)	0 (0%)
Background 1	27 (54%)	25 (50%)	13 (26%)	10 (20%)	22 (44%)	40 (80%)	1 (2%)
Background 2	21 (42%)	21 (42%)	16 (32%)	13 (26%)	18 (36%)	41 (82%)	1 (2%)
Background 3	23 (46%)	17 (34%)	18 (36%)	11 (22%)	17 (34%)	39 (78%)	1 (2%)
*P* value	0.61	0.36	0.76	0.78	0.70	0.95	0.80
Observational prospective with no risk intervention (e.g., soothing music)							
No background	19 (38%)	27 (54%)	12 (24%)	13 (26%)	17 (34%)	44 (88%)	1 (2%)
Background 1	20 (40%)	28 (56%)	13 (26%)	13 (26%)	19 (38%)	44 (88%)	2 (4%)
Background 2	18 (36%)	25 (50%)	16 (32%)	8 (16%)	18 (36%)	44 (88%)	1 (2%)
Background 3	23 (46%)	22 (44%)	16 (32%)	9 (18%)	19 (38%)	41 (82%)	1 (2%)
*P* value	0.76	0.64	0.74	0.48	0.97	0.76	0.89
Observational prospective with minimal risk intervention (e.g., measuring blood pressure)							
No background	25 (50%)	20 (40%)	17 (34%)	9 (18%)	14 (28%)	41 (82%)	1 (2%)
Background 1	23 (46%)	21 (42%)	13 (26%)	9 (18%)	21 (42%)	40 (80%)	2 (4%)
Background 2	26 (52%)	17 (34%)	17 (34%)	6 (12%)	16 (32%)	38 (76%)	1 (2%)
Background 3	25 (50%)	18 (36%)	15 (30%)	6 (12%)	16 (32%)	37 (74%)	1 (2%)
*P* value	0.94	0.84	0.79	0.70	0.49	0.76	0.89

Data as n (%). Statistical comparisons by Chi-square test

#### Survey 2

The primary outcome of the proportion of respondents who agree they felt overwhelmed on being approached for consent to observational research of no or minimal risk was 26 (52%; 95% CI 39-65%). Results suggest that being approached for consent to an observational research study is stressful, emotionally burdensome, or irritating for about half of the respondents (Table 3). Only 18% of respondents agreed that they “would feel excited about the opportunity” or “fear the risks” of participation in observational research (Table 3). Most respondents (from 60 to 74% depending on the question) answered that they understand “most of it” or “very well” the terms/concepts regarding observational research, including “no risk”, “minimal risk”, “consent bias”, “difficulties in obtaining consent”, “REB review and approval”, and “many safeguards required by the REB” (Table 4). However, after reading the background information defining/discussing these terms/concepts, most respondents (from 60 to 74% depending on the question) answered that their understanding had improved “a great deal” (from 4 to 5 on the Likert scale) (Table 4). Only 14–18% responded that their understanding had improved “not at all” (from 1 to 2 on the Likert scale). When asked “do you think the information given in this survey will influence your decision to have your child participate in an observational research study if asked in the future?”, responses were yes for 22 (44%) and no for 26 (52%).

**Table 3:** Survey 2: feelings about being approached for an observational research study

Question	Strongly Disagree	Disagree	Neither	Agree	Strongly Agree
I would feel stressed about being approached for this research study	7 (14%)	11 (22%)	9 (18%)	17 (34%)	6 (12%)
I would feel stressed about having to make a decision regarding my child’s participation in this research study	7 (14%)	17 (34%)	4 (8%)	17 (34%)	5 (10%)
I would feel excited about the opportunity to have my child participate in a research study	7 (14%)	9 (18%)	25 (50%)	5 (10%)	4 (8%)
I would fear the risks of participation in the research study	17 (34%)	14 (28%)	10 (20%)	6 (12%)	3 (6%)
I would feel too emotionally burdened to make the decision	5 (10%)	10 (20%)	10 (20%)	19 (38%)	6 (12%)
I would feel too overwhelmed to make the decision about my child’s participation at that time	5 (10%)	12 (24%)	7 (14%)	16 (32%)	10 (20%)
I would feel irritated about being asked to participate in a research study at this time	8 (16%)	11 (22%)	11 (22%)	15 (30%)	5 (10%)

**Table 4:** Survey 2: understanding of terms regarding observational research both before and after reading background information

Question	Do not understand	Understand a little	Understand most of it	Understand very well	Improved understanding after background information: score 4–5/1–2
Do you understand what “observational research with no risk” means?	4 (8%)	11 (22%)	10 (20%)	25 (50%)	34 (68%) / 9 (18%)
Do you understand what “observational research with minimal risk” means?	9 (18%)	11 (22%)	10 (20%)	20 (40%)	35 (70%) / 9 (18%)
Do you understand that a ‘consent bias’ may occur and make research findings misleading? (i.e., findings do not apply to all patients)	5 (10%)	10 (20%)	19 (38%)	16 (32%)	30 (60%) / 7 (14%)
Do you understand there are many difficulties involved in obtaining consent for research?	7 (14%)	9 (18%)	14 (28%)	20 (40%)	33 (66%) / 8 (16%)
Do you understand that a Research Ethics Board reviews and approves all research studies before they may begin?	6 (12%)	10 (20%)	14 (28%)	20 (40%)	35 (70%) / 9 (18%)
Do you understand that many safeguards are required by the Research Ethics Board to protect your child’s privacy?	2 (4%)	11 (22%)	14 (28%)	23 (46%)	37 (74%) / 9 (18%)

#### Free text responses

For both surveys, the themes of free text responses were similar (all responses are shown in Additional file 1: Table S3). In survey 1, the most common reasons that the survey would influence future decisions about research participation were that respondents have a better understanding of the terms/concepts (8/23, 35%) or are more comfortable with research and being approached (8/23, 35%). The most common reasons that the survey would not influence future decisions about research participation was that respondents already always participate (9/23, 39%), or always want to discuss participation (8/23, 35%). In survey 2, the most common reason that the survey would influence future decisions about research participation was that respondents have a better understanding of the terms/concepts (9/17, 53%). The most common reasons that the survey would not influence future decisions about research participation were that respondents already always participate (9/23, 39%) or that the decision would depend on the circumstance (7/23, 30%).

#### Post hoc comparisons

We explored whether dichotomized demographic variables [age, sex, child age, education level, and prior approach for research] were associated with the primary outcomes. For survey 1, the response of some alternative to signed informed consent being acceptable was not statistically significantly associated with any demographic variable for all four types of research. For survey 2, the response that “I would feel too overwhelmed to make the decision about my child’s participation at that time” was not statistically significantly associated with any demographic variable. These results are shown in Additional file 1: Table S4.

## Discussion

In this study, we directly asked parents/guardians about their opinions and knowledge regarding the consent process for observational research in PICU, both before and after providing background information. The main findings include the following. First, background information provided did not change respondent opinions about which methods of consent to observational research were acceptable. Even so, the proportion of respondents accepting an alternative to signed informed consent to observational research after receiving all of the background information was high. Second, being approached for consent to an observational research study was often considered stressful, emotionally burdensome, or irritating for respondents. Third, although the majority initially answered that they understood “most of it” or “very well” the terms/concepts involved, after reading the background information, most answered that their understanding had improved “a great deal”. This suggests that, for most participants, initial understanding of the concepts was poor. Finally, about half of the respondents for both surveys thought the information would not influence their decision in the future if approached for the participation of their child in observational research. The most common reasons for this are on opposite extremes: either respondents already always participate, or respondents always want to discuss participation, and the decision will depend on the circumstance.

Others have described that surrogate decision makers asked for consent to research are in crisis and experience added emotional burden from the approach [2–5], and that many lack knowledge of the research process, specifically of safeguards to privacy, and independent ethics committee review [6, 7]. Some studies have also found that provision of some knowledge, in focus group “dialogue” or information, made little change in acceptance of consent models [6, 7]; however, if there were no substitute decision maker, or consent “would make this research too difficult to carry out”, alternative methods were often endorsed [13, 16]. Finally, altruism and a desire for medical progress are reasons that consent is given for research [4, 14]. We extend these descriptions by finding that: a) the same emotional burdens and knowledge gaps exist in guardians making decisions about observational research for children in the PICU; b) knowledge of the difficulties involved in obtaining consent, and the resultant consent bias or inability to carry out the research, is also likely poor for many; c) although knowledge provision did not change the acceptance of alternative models of consent, most respondents continued to agree that some alternative model was acceptable even for observational research with a minimal risk intervention; and d) a minority (about 20%) of respondents believed a complete discussion and guardian agreement, even for observational research involving only chart review, is always needed.

Contrary to our hypothesis, provision of information about REB approval and safeguards to confidentiality, difficulties in doing research and resultant consent bias or abandonment of the research study, and emotional burdens on decision makers when asked for consent did not change the acceptability of alternative models of consent. Potential explanations for this finding include: some guardians simply do not want even observational research done without their explicit permission; the information was not well understood by participants; or the information was not explicit enough. The information may have been too complex or too comprehensive; future studies should examine simpler wording, or giving less background information in each of a series of separate surveys, or a focus group strategy where discussion of the issues is allowed to clarify concepts. Conversely, future studies should examine giving more explicit information on the magnitude of effects to make the points more salient. For example, in a Canadian multicenter cross-sectional study, consent for participation in research was missed in 28.8% (due to research staff workload and availability) and infeasible due to operational reasons in 28.5% (often due to difficulty contacting a decision maker or no decision maker being available) of patients [14]. Research studies thus take much longer than planned or are abandoned, even though patients “deserve timely identification of beneficial, non-beneficial and harmful interventions” [1]. Consent bias has been shown to result in unpredictable, but systematic deviation of results from the underlying truth because of differences in age, sex, race, income, education, health status, risk factors for adverse outcomes, medical interventions, and patient outcomes between consenters and non-consenters [8–12]. Symptoms of anxiety and depression are already present in 62% and 38% of ICU family members [19], and being approached to make a consent to research decision is associated with a 48% prevalence of PTSD [5]. This may be because approach for consent to research participation “suggests that an important choice is being made, one that balances risks and benefits…” [2]. This may be misleading, given the rigorous safeguards imposed by REB approval. These points are particularly important for future studies to examine given the concerning finding that up to 20% of respondents answered that an alternative consent model was not acceptable for chart review studies.

We believe that a “broader definition of ‘protecting’ acutely ill patients” may be needed, tailoring consent models to the risks [1]; the question is whether “individual privacy is more important than the societal benefits of research” [9], of balancing improved confidentiality over improved health and use of public resources, particularly for observational research with REB-imposed safeguards. This, and other surveys, suggests that most consenters do so for altruistic reasons (to help future patients, to advance medical progress) [4, 14], and therefore most people endorse alternative methods of consent when signed informed consent is not practicable [13, 16]. It is “thoughtful decision making... on the need for mandatory consent” that is necessary [10], considering more than guardian autonomy in determining the patient’s best-interests when no or minimal risk observational research is done.

This study has some strengths. First, the survey development methodology was according to published standards [18]. The surveys included the most important themes identified by a literature review, and agreed on by expert reviewers. Second, this is the first survey we are aware of to directly ask decision makers their opinions about alternative models of consent to observational research both before and after provision of background information. This study also has limitations. First, the surveys were information dense, and this may have been too comprehensive or complex to ensure adequate understanding by respondents. Pilot testing of the survey (although limited to two parents), distribution at a time when the patient was stable, and the unlimited time allowed for completion may mitigate this concern to some extent. In addition, the free-text responses did not suggest respondents felt they could not understand the material, although this was not specifically asked. Second, the small sample size from one institution limits the generalizability of the results. The findings being compatible with previous research in adult ICU decision makers may mitigate this limitation. Third, many families were missed, usually due to short PICU stays without a guardian available during study screening times; this low response rate may have introduced a consent bias. Even so, of approached guardians, the cooperation rate was high at 85%. Fourth, the small incentive of $5 may have contributed to a desirability bias in responses. This is unlikely with this small incentive and likely outweighed by the effect of increasing response rates.

## Conclusions

Being approached for consent to observational research is often stressful, even overwhelming for parents of critically ill children. In addition, parental understanding of risk, difficulties in obtaining consent, consent bias, and REB safeguards is likely poor for most. Although background information provided did not change aggregate parental opinions about which methods of consent to observational research are acceptable, most agreed with some alternative method of consent for observational research even with a minimal risk intervention. Future studies should confirm these findings, test simplifying the information provided (perhaps provided in a series of simplified surveys or in a focus group format), and test more explicit and quantitative information about the stress involved in research consent decisions, the wide-spectrum of factors leading to consent bias, the severity of limitations on research by requiring signed informed consent, and discussion of a “broader definition of ‘protecting’ acutely ill patients” [1].

## Supplementary information

**Additional file 1: Table S1.** Literature review themes regarding adverse effects from the requirement for informed consent to observational intensive care unit research of no or minimal risk. **Table S2.** Introductory and background information provided to participants in the surveys. **Table S3.** All free text responses to the two surveys when asked whether the information in the survey will influence the decisions to have their child participate in an observational research study in the future. **Table S4.** Post-hoc associations between demographic variables and primary outcomes of the surveys.

**Additional file 2.** Survey 1 used in the study.

**Additional file 3.** Survey 2 used in the study.

## Data Availability

The datasets used and/or analyzed during the current study are available from the corresponding author on reasonable request.

## Abbreviations

PICU: Pediatric intensive care unit
REB: Research ethics board
REDCap: Research Electronic Data Capture
