# Supplementary material for: Parental opinions regarding consent for observational research of no or minimal risk in the pediatric intensive care unit
Source: J Intensive Care. 2019 Dec 16;7:60. doi: 10.1186/s40560-019-0411-3 (PMC6916229; doi:10.1186/s40560-019-0411-3)
Supplement: Supplementary file 1 — Additional file 1: Table S1. Literature review themes regarding adverse effects from the requirement for informed consent to observational intensive care unit research of no or minimal risk. Table S2. Introductory and background information provided to participants in the surveys. Table S3. All free text responses to the two surveys when asked whether the information in the survey will influence the decisions to have their child participate in an observational research study in the future. Table S4. Post-hoc associations between demographic variables and primary outcomes of the surveys. [file 40560_2019_411_MOESM1_ESM.pdf]

Parental opinions regarding consent for observational research of no or minimal risk in the pediatric intensive care unit

Authors: Jessica Hodson BSc, Christiana Garros BSc candidate, Jodie Jensen RN, Jonathan P Duff MD, Gonzalo Garcia Guerra MD, Ari R Joffe MD.

Journal: Journal of Intensive Care

Supplemental File 1 (pdf). Supplemental tables.

Table S1. Literature review themes regarding adverse effects from the requirement for informed consent to observational intensive care unit research of no or minimal risk.

Table S2. Introductory and background information provided to participants in the surveys.

Table S3. All free text responses to the two surveys when asked whether the information in the survey will influence the decisions to have their child participate in an observational research study in the future.

Table S4. Post-hoc associations between demographic variables and primary outcomes of the surveys.

Table S1. Literature review themes regarding adverse effects from the requirement for informed consent to observational intensive care unit research of no or minimal risk.

Theme	Details
Added stress on the decision-maker	Decision makers are often overwhelmed, emotionally burdened, and stressed given the severity of illness in their loved one. Asking for consent to research adds to this emotional burden.
	Many decision makers have said that they were too overwhelmed, stressed, and emotional to think about research decisions.[2-4]
	One study found that being asked to consent for research was independently associated with post-traumatic stress symptoms in decision makers.[5]
Lack of decision-maker knowledge about the research process and safeguards	Despite being given informed consent information, many decision makers had lack of knowledge of the safeguards present in the research process. They were not aware of de-identification, confidentiality, and privacy safeguards, including database security, anonymizing data, and no release of data to insurance or pharmaceutical companies.[6,7]
	Many were not aware of review by independent ethics committees.[6,7]
	For no or minimally risk studies, many still perceived that there was a high risk of harm to the patient, and conversely, many consent because they mistakenly think the study will benefit the participating patient (therapeutic misconception).[2]
The importance of consent [also known as authorization, volunteer, selection, response, refusal, participation] bias	Systematic reviews have found that the incidence of, and risk factors for a condition or finding can systematically differ between those consenting and not-consenting to observational research.[7-12]
	This leads to systematic error in the results, giving unreliable or misleading results of research, which can adversely affect the patient by having their care based on these misleading research results.
The resulting lack of research	The cost and administrative burden of obtaining informed consent can make research impractical, being too difficult to carry out. Often decision makers are unavailable (narrow time windows, difficulty contacting families, or no parent at bedside), and there are practical obstacles (including staff time, workload, and availability) to obtaining consent.[13,14]
	This can ultimately lead to lack of research, delayed research, and prolonged studies, thus impeding medical progress.[1,14]
	This amounts to weighing the balance of individual privacy and autonomy versus improved health for society and future patients.

Table S2. Introductory and background information provided to participants in the surveys.

Term	Description
**Survey 1 Introductory Information**	
Observational study	The study involves no change in your child’s care; it simply records information from observation alone (i.e., no invasive treatment intervention). Four types of observational studies are (in order of increasing potential ‘risk’).
Observational retrospective	A retrospective study looks backwards at past information. This is done through chart review (i.e., reading information that is already in the medical record, usually after the patient has been discharged from hospital).
Observational prospective	A prospective study involves patients who *currently* have a certain condition. This is done through chart review (i.e., recording information that is *currently* being put in the medical record, often while the patient is still in hospital).
With no risk intervention	An observational prospective study where an intervention that is thought to be of no risk to the patient is done. An example of “no risk” is playing soothing music at the bedside of the patient.
With a minimal risk intervention	An observational prospective study where an intervention with minimal risk to the patient is done. “Minimal risk” is defined as having no possibility of harm greater than encountered in everyday life. An example of a “minimal risk” intervention is measuring a cuff blood pressure on the arm.
Signed informed consent	You give consent in writing before the study procedures can begin.
Opt-out	For each study, you are asked verbally if you object to participation, and if you do not say you object, your child is included in the research study. For example, you might be informed of the study during patient rounds, and have the chance to object at that time.
Physicians’ consent	Two physicians, one of whom is the attending physician, decide whether to consent for your child to be included in the research study.
Broad authorization	Signed informed consent is given by you on admission to the hospital for your child to participate in all observational research that is of no or minimal risk
Waived	There is no requirement for consent for your child to be included in the research study.
**Survey 1 Background information**	
Background 1: REB approval and confidentiality safeguards	In order for a research study to begin it must first be reviewed and approved by a University Research Ethics Board, and found to be safe and ethical for all patients involved. In order to protect the confidentiality of all participants many safeguards are required by the Research Ethics Board. These safeguards include making all databases anonymous and keeping any potentially identifying information (e.g., name, date of birth) confidential. All databases used during the research process are secure and inaccessible to outside sources such as insurance or pharmaceutical companies.

Background 2: Stress of being approached for research	Many research studies have found that a main reason why parents or guardians do not give consent for research while their child is in the hospital is because they are too overwhelmed and stressed to think about giving consent at that time. Many parents have said that being approached for consent for a research study during this time of crisis adds extra stress and emotional burden. In fact, parents often tell us they are unable to remember much of the information they received about their child in the first days in the PICU.
Background 3: Process difficulties in obtaining consent, and consent bias	The requirement for signed informed consent can affect research findings in many ways. First, approaching parents for consent is time consuming and therefore costly and sometimes not possible. For example, parents may not be present at the bedside when research staff are working or when the study should start. Sometimes this can make the study impractical, and therefore the research may not be done. This means than any potential benefits and resulting medical progress may not occur. Second, if some parents do not consent, something called "consent bias" can occur. This means that if there is a difference between children whose parents do consent for the study and children whose do not, then the study results can be misleading (i.e., not apply to all patients).
**Survey 2 Background information**	
Background 1: Observational studies	Observational study: means that the study involves no change in your child's care; the study records information from observation alone (i.e., no treatment intervention). Two types of observational study are: 1. Observational with no risk intervention: an observational study where an intervention that is thought to be of no risk to the patient is done. An example of "no risk" is playing soothing music at the bedside of the patient; 2. Observational with minimal risk intervention: an observational study where an intervention with minimal risk is given to all participating patients. "Minimal risk" is defined as having no possibility of harm greater than encountered in everyday life. An example of "minimal risk" intervention is measuring a cuff blood pressure on the arm of the patient.
Background 2: consent bias and difficulty of research	Consent bias: If some parents do not consent, something called a 'consent bias' can occur. This means that if there is a difference between the children of parents who do not consent for the study and the children of those who do consent, then the study's results can be misleading (i.e., not apply to all patients).<br>Difficulty in obtaining consent: Approaching parents for consent is time consuming and therefore costly and sometimes not possible. For example, parents may not be present at the bedside when research staff are working or when the study should start. Requiring consent can make the study impractical, and therefore the research may not be done. This means that any potential benefits and resulting medical progress may not occur.
Background 3: REB process and safeguards	Research Ethics Board: before a research study can begin it must first be reviewed and approved by a University Research Ethics Board whose members decide if the study is safe and ethical for all patients involved.<br>Research safeguards: In order to protect the confidentiality of all participants many safeguards are required by the Research Ethics Board. These safeguards include making all databases anonymous and keeping any potentially identifying information (e.g., name, date of birth) confidential. All databases used during the research process are secure and inaccessible to outside sources such as insurance or pharmaceutical companies.

Table S3. All free text responses to the two surveys when asked whether the information in the survey will influence the decisions to have their child participate in an observational research study in the future.

Theme	Response was "yes"; question "in what way?"	Response was "no"; question "why not?"
**Survey 1**		
Always participate anyways	"I believe studies are the answer"<br>"I have always agreed…"<br>"Research is important in our healthcare system."	"I would have allowed the study before…"<br>"We already have no issues allowing…"<br>"We are fine with all observational studies…"<br>"Pretty willing to studies already…"<br>"I would say yes to most studies regardless"<br>"I'd participate anyway"<br>"I have always believe in the importance of research"<br>"Would have given consent regardless"<br>"I always agree to participating"
Have a better understanding vs. already understood	"Understanding 'consent bias' now, I think a broad authorization would assist…"<br>"I now have more understanding of the work…"<br>"It gave more clarification about the consents…"<br>"I now understand that there is more than one way to consent"<br>"Knowing the types of consent made us aware on how to respond…"<br>"It was very informative…"<br>"The parents will have a better understanding of why research is important…"<br>"The information provided was very informative and knowing confidentiality is always top priority."	"Already aware of most of the information given."
Are more comfortable with research and being approached vs. always want discussion	"I think that research such as this can help children in the future"<br>"To help other patients to cooperate well in all procedures"<br>"If they were to know about research ahead of time and sign off on it, they would not feel it is an	"Would not be interested. Would have too much on our mind"<br>"Wish consent of parents always"<br>"I always like to know who is accessing my child's info, and for what reasons"<br>"I want to be informed or at the very least aware of

	annoyance or frustration…"<br>"For the better for child in health"<br>"Because this will help in future patients…"<br>"It may help someone in the future"<br>"I would think it's the least we could do, and perhaps help someone else's child."<br>"Only the 'consent' for major decisions would have to be addressed, cutting down on information overload…"	everything that affects their lives…"<br>"As long as signed consent is always obtained"<br>"But I'd like to be asked"<br>"I would always like to be involved in the discussion"<br>"I expect I would be given all pertinent information at the time"
It depends on the study	"As long as it's the right time and not stressful"<br>"But it may also depend on the situation at the time"<br>"Depending on how we look at it from different perspectives"	"Will always be dependent upon the individual study"<br>"I do not know how I'll react until it occurs"<br>"I would base my decision on what the study is"
Survey will not be remembered	"The survey questions are too complicated (confusing)…"	"Too much to try to remember"<br>"Won't remember the survey enough"
**Survey 2**		
Always participate anyways	"I always participate"<br>"Always willing to help out"<br>"I already participate in them as I would like to see medical advancements"	"Always been willing to participate"<br>"Would have agreed anyways"<br>"I am always open to participating"<br>"I would give consent either with or without the survey"<br>"We would agree anyways"<br>"We are already very supportive of research studies"<br>"If my child's journey could help others I am happy to help"<br>"I would have allowed it either way"<br>"I was OK with it already"
Have a better understanding vs. already understood	"I know a little more what is involved"<br>"Just more so timing of asking to do study when you are in crisis"<br>"You actually know the terms and meanings"<br>"Knowing what the terms mean and understanding that results are more accurate…"	"I was already aware of this information"<br>"Already feel I knew majority of information"<br>"I already have a good understanding"

	"Because you have already questioned to find out if its worth it or not"<br>"The better understanding everyone gains the more care and quicker healing can occur"<br>"I feel more informed about the safeguards that are in place. I can also appreciate how difficult it would be to get consent when parents are in shock…"<br>"Now I have a better understanding about how research surveys are conducted."<br>"I'm well aware of the troubles on getting data needed for research."	
Are more comfortable with research and being approached vs. will be too stressed/say no	"I may be more comfortable to let her participate"<br>"If we could help a child avoid this, we would"<br>"Understanding that it is strictly observational makes me feel more comfortable even in a stressful time"	"This is extremely stressful in an already excruciatingly stressful situation"<br>"We have already had a negative experience with research study"<br>"We already took the decision of not giving the permission of our baby for research"<br>"I may still be stressed and emotional at the time"
It depends on the study	"It will matter to the facts- if it improves quality of life or keeps her alive"<br>"Depending on the situation"	"Depending on the nature of my child's situation"<br>"I would solely base my decision on my child's state at the time of being asked"<br>"It fully depends on the research topic"<br>"It will depend entirely on what the study is about"<br>"The decision will be based on the current conditions of the scenario"<br>"Depends on the circumstance"<br>"Depends on our situation"

Table S4. Post-hoc associations between demographic variables and primary outcomes of the surveys.

Outcome	Age	p-value	Sex	p-value	Child age	p-value	Education	p-value	Prior Approach	p-value
**Survey #1: Any alternative [after all background information]**										
Observational retrospective	27/30 vs 12/18	0.06	10/15 vs 29/34	0.25	17/22 vs 22/27	0.74	19/22 vs 20/27	0.48	20/25 vs 19/24	0.99
Observational Prospective	26/30 vs 12/18	0.15	10/15 vs 28/34	0.28	17/22 vs 21/27	0.99	18/22 vs 20/27	0.73	20/25 vs 18/24	0.74
Observational prospective with no risk intervention	25/30 vs 15/18	0.99	11/15 vs 29/34	0.43	18/22 vs 22/27	0.99	19/22 vs 21/27	0.49	23/25 vs 17/24	0.07
Observational prospective with minimal risk intervention	23/30 vs 13/18	0.74	9/15 vs 27/34	0.18	16/22 vs 20/27	0.99	15/22 vs 21/27	0.53	20/25 vs 16/24	0.35
**Survey #2: "I would feel too overwhelmed to make the decision about my child's participation at that time"**										
SA/A vs. SD/D/N	17/28 vs. 9/22	0.25	5/12 vs 21/37	0.51	11/23 vs 14/26	0.78	13/22 vs 13/28	0.41	16/30 vs 10/20	0.99

Statistical testing done using Fisher Exact test. SA: strongly agree; A: agree; N: neither; D: disagree; SD: strongly disagree.
