# Supplementary material for: Parental opinions regarding consent for observational research of no or minimal risk in the pediatric intensive care unit
Source: J Intensive Care. 2019 Dec 16;7:60. doi: 10.1186/s40560-019-0411-3 (PMC6916229; doi:10.1186/s40560-019-0411-3)
Supplement: Supplementary file 2 — Additional file 2. Survey 1 used in the study. [file 40560_2019_411_MOESM2_ESM.docx]

**Parental opinions regarding consent for observational research of no or minimal risk in the pediatric intensive care unit**

**Authors:** Jessica Hodson BSc, Christiana Garros BSc candidate, Jodie Pugh RN, Jonathan P Duff MD, Gonzalo Garcia Guerra MD, Ari R Joffe MD.

**Journal:** Journal of Intensive Care

**Supplemental File 2 (pdf).** Survey 1.


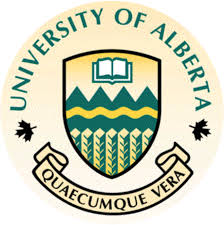
PARENTAL OPINIONS REGARDING CONSENT FOR OBSERVATIONAL RESEARCH

WE WANT TO KNOW

HOW DO YOU FEEL RESEARCHERS SHOULD ASK FOR CONSENT FOR YOUR CHILD’S INVOLVMENT IN AN OBSERVATIONAL STUDY?

***WHY THE SURVEY?***

As a group of Intensive Care Medicine physicians at the University of Alberta who are actively involved in research, we believe it is critical to know the public’s thoughts on this issue.

***VOLUNTARY PARTICIPATION***

- This survey has been approved by the University’s Health and Research Ethic’s Board. If you have concerns, please contact **(780) 492-0302.**
- You are under no obligation to complete this survey, especially if you find the subject matter unpleasant.
- You may leave any question you find unpleasant unanswered and still return the remainder of the survey.
- Should you return a completed survey, please note this implies consent to participate.
- Your responses are voluntary and will be kept confidential. The results will be recorded anonymously and will only be described in aggregate. Study information will be stored securely by investigators for 7 years, as required by Canadian law.
- We anticipate the results of this survey to benefit medical knowledge in a general way; thus, there is no direct benefit or risk to you or your child from participation.

WHAT YOU SHOULD DO NOW

PLEASE TAKE 15-20 MINUTES TO FILL OUT THE SURVEY

YOU MAY RETURN THE COMPLETED SURVEY TO THE BEDSIDE NURSE

ENJOY A COFFEE ON US!


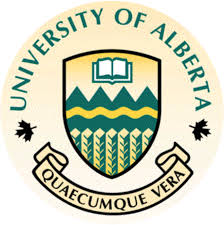
PREAMBLE TO THE STUDY

In this survey you will be given a hypothetical scenario. You will then be asked to give your opinion regarding how you think different types of research studies should obtain consent for your child’s participation.

TYPES OF RESEARCH

For the purpose of the study you will need to be aware of the differences between 4 types of **observational** research studies:

- **Observational study**: the study involves no change in your child’s care; it simply records information from observation alone (i.e., no invasive treatment intervention). Four types of observational studies are (in order of increasing potential ‘risk’):
- **1. Observational retrospective:** a retrospective study looks backwards at past information. This is done through chart review (i.e., reading information that is already in the medical record, usually after the patient has been discharged from hospital).
- **2. Observational prospective:** a prospective study involves patients who *currently* have a certain condition. This is done through chart review (i.e., recording information that is *currently* being put in the medical record, often while the patient is still in hospital).
- **3. Observational prospective with a no risk intervention:** an observational prospective study where an intervention that is thought to be of no risk to the patient is done. An example of “no risk” is playing soothing music at the bedside of the patient.
- **4. Observational prospective with a minimal risk intervention:** an observational prospective study where an intervention with minimal risk to the patient is done. “Minimal risk” is defined as having no possibility of harm greater than encountered in everyday life. An example of a “minimal risk” intervention is measuring a cuff blood pressure on the arm.

TYPES OF CONSENT

For the purpose of the study you will need to be aware of 5 different ways consent for an observational research study may be obtained:

- **Signed informed:** you give consent in writing before the study procedures can begin.
- **Opt-out:** for each study, you are asked verbally if you object to participation, and if you do not say you object, your child is included in the research study. For example, you might be informed of the study during patient rounds, and have the chance to object at that time.
- **Physicians:** two physicians, one of whom is the attending physician, decide whether to consent for your child to be included in the research study.
- **Broad authorization:** signed informed consent is given by you on admission to the hospital for your child to participate in all observational research that is of no or minimal risk.
- **Waived:** there is no requirement for consent for your child to be included in the research study.

ALL QUESTIONS WILL REFER TO THIS SCENARIO:

Your child has been in a car crash and has been admitted to the Stollery Children’s Hospital Pediatric Intensive Care Unit (PICU). Currently, your child is in critical condition and on a ventilator (a breathing machine) to help with breathing. There is an observational research study being conducted in the PICU. In your opinion, how should the consent to participate in this observational research study for your child be obtained?

***Listed below are the 4 types of observational research studies previously described. Please select which method(s) you believe would be acceptable to obtain consent by marking the box with an X. You should select all the methods that would be acceptable to you.***

|  | **Signed informed consent** | **Opt-out consent** | **Physicians consent** | **Waived consent** | **Broad authorization consent** |
| --- | --- | --- | --- | --- | --- |
| **Observational retrospective (e.g. chart review)** |  |  |  |  |  |
| **Observational prospective (e.g. chart review)** |  |  |  |  |  |
| **Observational prospective with no risk intervention (e.g. soothing music)** |  |  |  |  |  |
| **Observational prospective with minimal risk intervention (e.g. measuring blood pressure)** |  |  |  |  |  |

BACKGROUND INFORMATION #1

In order for a research study to begin it must first be reviewed and approved by a University Research Ethics Board, and found to be safe and ethical for all patients involved. In order to protect the confidentiality of all participants many safeguards are required by the Research Ethics Board. These safeguards include making all databases anonymous and keeping any potentially identifying information (e.g., name, date of birth) confidential. All databases used during the research process are secure and inaccessible to outside sources such as insurance or pharmaceutical companies.

|  | **Signed informed consent** | **Opt-out consent** | **Physicians consent** | **Waived consent** | **Broad authorization consent** |
| --- | --- | --- | --- | --- | --- |
| **Observational retrospective (e.g., chart review)** |  |  |  |  |  |
| **Observational prospective (e.g., chart review)** |  |  |  |  |  |
| **Observational prospective with no risk intervention (e.g., soothing music)** |  |  |  |  |  |
| **Observational prospective with minimal risk intervention (e.g., measuring blood pressure)** |  |  |  |  |  |

***With this information in mind, please select which method(s) you believe would be acceptable to obtain consent by marking the box with an X. You should select all the methods that would be acceptable to you.***

|  | **Signed informed consent** | **Opt-out consent** | **Physicians consent** | **Waived consent** | **Broad authorization consent** |
| --- | --- | --- | --- | --- | --- |
| **Observational retrospective (e.g., chart review)** |  |  |  |  |  |
| **Observational prospective (e.g., chart review)** |  |  |  |  |  |
| **Observational prospective with no risk intervention (e.g., soothing music)** |  |  |  |  |  |
| **Observational prospective with minimal risk intervention (e.g., measuring blood pressure)** |  |  |  |  |  |

***With this information in mind, please select which method(s) you believe would be acceptable to obtain consent by marking the box with an X. You should select all the methods that would be acceptable to you.***

BACKGROUND INFORMATION #2

Many research studies have found that a main reason why parents or guardians do not give consent for research while their child is in the hospital is because they are too overwhelmed and stressed to think about giving consent at that time. Many parents have said that being approached for consent for a research study during this time of crisis adds extra stress and emotional burden. In fact, parents often tell us they are unable to remember much of the information they received about their child in the first days in the PICU.

***With this information in mind, please select which method(s) you believe would be acceptable to obtain consent by marking the box with an X. You should select all the methods that would be acceptable to you.***

BACKGROUND INFORMATION #3

The requirement for signed informed consent can affect research findings in many ways. First, approaching parents for consent is time consuming and therefore costly and sometimes not possible. For example, parents may not be present at the bedside when research staff are working or when the study should start. Sometimes this can make the study impractical, and therefore the research may not be done. This means than any potential benefits and resulting medical progress may not occur. Second, if some parents do not consent, something called “consent bias” can occur. This means that if there is a difference between children whose parents do consent for the study and children whose do not, then the study results can be misleading (i.e., not apply to all patients).

|  | **Signed informed consent** | **Opt-out consent** | **Physicians consent** | **Waived consent** | **Broad authorization consent** |
| --- | --- | --- | --- | --- | --- |
| **Observational retrospective (e.g., chart review)** |  |  |  |  |  |
| **Observational prospective (e.g., chart review)** |  |  |  |  |  |
| **Observational prospective with no risk intervention (e.g., soothing music)** |  |  |  |  |  |
| **Observational prospective with minimal risk intervention (e.g., measuring blood pressure)** |  |  |  |  |  |

***Do you think the information given in this survey will influence your decision to have your child participate in an observational research study if asked in the future?***

- YES
  - If so, in what way? _______________________________________________________________________________________________________________________________________________________________
- NO
  - Why not?

______________________________________________________ ______________________________________________________ ______________________________________________________

DEMOGRAPHIC INFORMATION

***Please select the most appropriate description of yourself from each section below***

***Your Age (years)***

- 18 – 24
- 25 – 34
- 35 – 44
- 45 and older

***Sex***

- Male
- Female

***Age of your child (years)***

- Under 2
- 2 – 6
- 7 – 11
- 12 and older

***Highest Level of Education Completed***

- Did not complete high school
- High school completed
- At least one year of post-secondary completed
- Post-secondary degree/diploma obtained

***Work in the Field of Medicine & Health***

- I **have/do not** work in healthcare
- I **have/do** work in the healthcare field
  - Physician
  - Nurse
  - Other work in healthcare (specify) _________________

***I have been approached to have my child participate in a research study during this or any other previous hospitalization***

- Yes, and I **did** give consent to participate in the study
- Yes, and I **did not** give consent to participate in the study
- No, I have never been approached to have my child participate in a research study before
