# Supplementary material for: Parental opinions regarding consent for observational research of no or minimal risk in the pediatric intensive care unit
Source: J Intensive Care. 2019 Dec 16;7:60. doi: 10.1186/s40560-019-0411-3 (PMC6916229; doi:10.1186/s40560-019-0411-3)
Supplement: Supplementary file 3 — Additional file 3. Survey 2 used in the study. [file 40560_2019_411_MOESM3_ESM.docx]

**Parental opinions regarding consent for observational research of no or minimal risk in the pediatric intensive care unit**

**Authors:** Jessica Hodson BSc, Christiana Garros BSc candidate, Jodie Pugh RN, Jonathan P Duff MD, Gonzalo Garcia Guerra MD, Ari R Joffe MD.

**Journal:** Journal of Intensive Care

**Supplemental File 3 (pdf).** Survey 2.


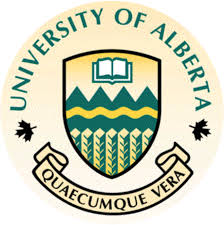
PARENTAL OPINIONS REGARDING CONSENT FOR OBSERVATIONAL RESEARCH

WE WANT TO KNOW

HOW WELL DO YOU UNDERSTAND WHAT IS INVOLVED IN OBSERVATIONAL RESEARCH OF NO TO MINIMAL RISK?

***WHY THE SURVEY?***

As a group of Intensive Care Medicine physicians at the University of Alberta who are actively involved in this discussion, we believe it is critical to know the public’s thoughts on this issue.

***VOLUNTARY PARTICIPATION***

- This survey has been approved by the University’s Health and Research Ethic’s Board. If you have concerns, please contact **(780) 492-0302.**
- You are under no obligation to complete this survey, especially if you find the subject matter unpleasant.
- You may leave any question you find unpleasant unanswered and still return the remainder of the survey.
- Should you return a survey, please note this implies consent to participate.
- Your responses are voluntary and will be kept confidential. The results will be recorded anonymously and will only be described in aggregate. Study information will be stored securely by investigators for 7 years, as required by Canadian law.
- We anticipate the results of this survey to benefit medical knowledge in a general way; thus, there is no direct benefit or risk to you or your child from participation.

WHAT YOU SHOULD DO NOW

PLEASE TAKE 10-15 MINUTES TO FILL OUT THE SURVEY

YOU MAY RETURN THE COMPLETED SURVEY TO THE BEDSIDE NURSE

ENJOY A COFFEE ON US!


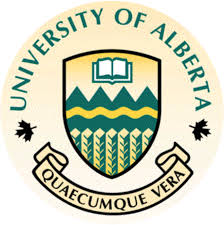
PREAMBLE TO THE STUDY

In this survey you will be given a hypothetical scenario. You will then be asked to give your opinion regarding how you believe you would feel in this situation. Next, we will ask about your knowledge regarding the consent process for observational research of no or minimal risk.

ALL QUESTIONS WILL REFER TO THIS SCENARIO:

Your child has been in a car crash and has been admitted to the Stollery Children’s Hospital Pediatric Intensive Care Unit (PICU). Currently, your child is in critical condition and on a ventilator (breathing machine) to help with breathing. There is a research study being conducted at the PICU that involves patients after a car crash. The study consists of recording information that is currently being put in the medical record and measuring a cuff blood pressure on your child’s arm.

PART ONE

***Imagining you are in this scenario, how do you think you would feel about being approached for this research study? Please circle the best response to each question.***

| ***Question*** | ***Strongly Disagree*** | ***Disagree*** | ***Neither Agree nor Disagree*** | ***Agree*** | ***Strongly Agree*** |
| --- | --- | --- | --- | --- | --- |
| I would feel stressed about being approached for this research study | 1 | 2 | 3 | 4 | 5 |
| I would feel stressed about having to make a decision regarding my child’s participation in this research study | 1 | 2 | 3 | 4 | 5 |
| I would feel excited about the opportunity to have my child participate in a research study | 1 | 2 | 3 | 4 | 5 |
| I would fear the risks of participation in the research study | 1 | 2 | 3 | 4 | 5 |
| I would feel too emotionally burdened to make the decision | 1 | 2 | 3 | 4 | 5 |
| I would feel too overwhelmed to make the decision about my child’s participation at that time | 1 | 2 | 3 | 4 | 5 |
| I would feel irritated about being asked to participate in a research study at this time | 1 | 2 | 3 | 4 | 5 |

***Based on your current knowledge, how well do you think you understand the following concepts related to research? Please circle the best response to each question.***

| ***Questions*** | ***Do not understand*** | ***Understand a little*** | ***Understand most of it*** | ***Understand very well*** |
| --- | --- | --- | --- | --- |
| Do you understand what “observational research with no risk” means? | 1 | 2 | 3 | 4 |
| Do you understand what “observational research with minimal risk” means? | 1 | 2 | 3 | 4 |
| Do you understand that a ‘consent bias’ may occur and make research findings misleading? (i.e., findings do not apply to all patients) | 1 | 2 | 3 | 4 |
| Do you understand there are many difficulties involved in obtaining consent for research? | 1 | 2 | 3 | 4 |
| Do you understand that a Research Ethics Board reviews and approves all research studies before they may begin? | 1 | 2 | 3 | 4 |
| Do you understand that many safeguards are required by the Research Ethics Board to protect your child’s privacy? | 1 | 2 | 3 | 4 |

PART TWO.

**BACKGROUND INFORMATION #1**

**Observational study:** means that the study involves **no** change in your child’s care; the study records information from observation alone (i.e., no treatment intervention). Two types of observational study are:

**1. Observational with no risk intervention:** an observational study where an intervention that is thought to be of no risk to the patient is done. An example of “no risk” is playing soothing music at the bedside of the patient.

**2. Observational with minimal risk intervention:** an observational study where an intervention with minimal risk is given to all participating patients. “Minimal risk” is defined as having no possibility of harm greater than encountered in everyday life. An example of “minimal risk” intervention is measuring a cuff blood pressure on the arm of the patient.

***Did reading the background information above improve your understanding of the following concepts? Please circle the best response to each question.***

|  | Not at all | | Somewhat | A great deal | |
| --- | --- | --- | --- | --- | --- |
| Understanding of what “observational research with no risk” means? | 1 | 2 | 3 | 4 | 5 |
| Understanding of what “observational research with minimal risk” means? | 1 | 2 | 3 | 4 | 5 |

**BACKGROUND INFORMATION #2**

**Consent bias:** If some parents do not consent, something called a ‘consent bias’ can occur. This means that if there is a difference between the children of parents who do not consent for the study and the children of those who do consent, then the study’s results can be misleading (i.e., not apply to all patients).

**Difficulty in obtaining consent:** Approaching parents for consent is time consuming and therefore costly and sometimes not possible. For example, parents may not be present at the bedside when research staff are working or when the study should start. Requiring consent can make the study impractical, and therefore the research may not be done. This means that any potential benefits and resulting medical progress may not occur.

***Did reading the background information above improve your understanding of the following concepts? Please circle the best response to each question.***

|  | Not at all | | Somewhat | A great deal | |
| --- | --- | --- | --- | --- | --- |
| Understanding that consent bias could occur and make research findings less reliable? | 1 | 2 | 3 | 4 | 5 |
| Understanding that there are many difficulties in obtaining consent? | 1 | 2 | 3 | 4 | 5 |

**BACKGROUND INFORMATION #3**

**Research Ethics Board:** before a research study can begin it must first be reviewed and approved by a University Research Ethics Board whose members decide if the study is safe and ethical for all patients involved.

**Research safeguards:** In order to protect the confidentiality of all participants many safeguards are required by the Research Ethics Board. These safeguards include making all databases anonymous and keeping any potentially identifying information (e.g., name, date of birth) confidential. All databases used during the research process are secure and inaccessible to outside sources such as insurance or pharmaceutical companies.

***Did reading the background information above improve your understanding of the following concepts? Please circle the best response to each question.***

|  | Not at all | | Somewhat | A great deal | |
| --- | --- | --- | --- | --- | --- |
| Understanding that a Research Ethics Board reviews and approves all research studies before they begin? | 1 | 2 | 3 | 4 | 5 |
| Understanding that many safeguards are required by the Research Ethics Board to protect your child’s privacy? | 1 | 2 | 3 | 4 | 5 |

***Do you think the information given in this survey will influence your decision to have your child participate in an observational research study if asked in the future?***

- YES
  - If so, in what way? _______________________________________________________________________________________________________________________________________________________________
- NO
  - Why not?

DEMOGRAPHIC INFORMATION

***Please select the most appropriate description of yourself from each section below***

***Age (years)***

- 18 – 24
- 25 – 34
- 35 – 44
- 45 and older

***Sex***

- Male
- Female

***Age of your child (years)***

- Under 2
- 2 – 6
- 7 – 11
- 12 and older

***Highest Level of Education Completed***

- Did not complete high school
- High school completed
- At least one year of post-secondary completed
- Post-secondary degree/diploma obtained

***Work in the Field of Medicine & Health***

- I **have/do not** work in healthcare
- I **have/do** work in the healthcare field
  - Physician
  - Nurse
  - Other work in healthcare (specify) _________________

***I have been approached to have my child participate in a research study during this or any other previous hospitalization***

- Yes, and I **did** give consent to participate in the study
- Yes, and I **did not** give consent to participate in the study
- No, I have never been approached to have my child participate in a research study before
